# Additive effects of active vitamin D3 or vitamin E administration on therapeutic lower-limb home exercise for knee osteoarthritis: A pilot prospective randomized controlled trial

**DOI:** 10.1097/MD.0000000000051197

**Published:** 2026-10-16

**Authors:** Hiroaki Kanazawa, Hisashi Kurosawa, Masashi Nagao, Masato Koike, Kei Miyagawa, Yu Ozaki, Yoshiyuki Iwase, Muneaki Ishijima

**Affiliations:** a Department of Orthopaedics, Juntendo University Faculty of Medicine, Bunkyo-ku, Tokyo, Japan; b Department of Orthopaedic Surgery, Juntendo University, Tokyo Koto Geriatric Medical Center, Koto-ku, Tokyo, Japan; c Department of Orthopaedic Surgery, Medical Technology Innovation Center, Clinical Research & Trial Center, Graduate School of Health and Sports Science, Juntendo University, Bunkyo-ku, Tokyo, Japan; d Department of Regenerative Medicine for Locomotive Organ, Faculty of Medicine, Juntendo University, Tokyo, Japan.

**Keywords:** active vitamin D3, home exercise, knee osteoarthritis, randomized controlled pilot trial, vitamin E

## Abstract

**Background:**

Quadriceps weakness, inflammation, and reduced physical function are common in knee osteoarthritis (OA), and lower limb exercise therapy is an established non-pharmacological treatment. Vitamin D3 (vit D3) and vitamin E (vit E) have been proposed as adjunctive therapies; however, clinical findings are inconsistent. We aimed to examine whether combining lower limb home exercises with oral active vit D3 or vit E supplementation provides additive benefits compared with home exercise alone in women with symptomatic knee OA.

**Methods:**

Eighty-eight postmenopausal women with knee OA (Kellgren–Lawrence grade 2–4; 71.9 ± 5.6 years) were randomly assigned to receive therapeutic lower limb home exercise only, home exercise with active vit D3 administration, or home exercise with vit E administration. Participants performed isometric lower limb exercises twice daily for 24 weeks. Subsequently, they were assessed using the Japanese knee osteoarthritis measure (JKOM) as the primary outcome. Secondary outcomes were short-form health survey, grip strength, quadriceps muscle strength, 10-m walk test, inflammatory markers, bone mineral density, and body composition. For data analyses, we used paired and independent *t* tests, analysis of variance, and analysis of covariance, adjusting for age, body mass index, and Kellgren–Lawrence grade.

**Results:**

Eighty participants completed follow-up. All groups showed significant improvements in JKOM score, quadriceps muscle strength, and 10-m walk test. Grip strength improved significantly only in the group that received active vit D. No significant between-group differences were observed in JKOM scores, short-form health survey-36, muscle strength, or physical performance. Inflammatory markers decreased significantly in the vit E group, although between-group differences were not observed. No other significant between-group differences were found, except in bone mineral density.

**Conclusions:**

Administering active vit D3 or vit E for 24 weeks as an adjunct to lower limb home exercises did not yield significant additive effects on symptoms, muscle strength, or physical performance in women with knee OA. Exercise therapy alone remained effective, and vitamin supplementation showed nutrient-specific trends.

## 1. Introduction

Osteoarthritis (OA) is a leading cause of musculoskeletal pain and disability worldwide and is involved in articular cartilage degradation, synovial inflammation, joint space narrowing, subchondral bone changes, and osteophyte formation.^[1]^ Symptoms typically include joint pain, muscle weakness, and reduced mobility, which can result in significant disability, particularly in older individuals.^[2]^ Despite its prevalence, effective preventive and therapeutic treatments remain limited, with current management primarily focusing on symptom relief.

Quadriceps muscle weakness is frequently observed in knee OA, regardless of knee pain or muscular atrophy.^[3]^ The quadriceps are crucial for functional tasks, such as standing, stair climbing, and walking,^[4]^ making strengthening a key component in OA management.^[5]^ According to Osteoarthritis Research Society International guidelines, lower limb exercise therapy is the most evidence-based non-pharmacological treatment for OA.^[6]^

Although exercise plays a critical role in OA management, pharmacological treatments are commonly used. However, no disease-modifying therapies for OA currently exist, highlighting the need for cost-effective treatments to prevent or slow disease progression.^[7]^ Among emerging pharmacological approaches, nutritional supplements have gained attention. Vitamin D (vit D) is vital for numerous biological processes, including bone growth and remodeling, immune function, and cartilage and muscle health.^[8]^ A large-scale prospective study conducted by UK Biobank showed that serum vit D levels are associated with the risk of developing OA via markers of cartilage metabolism.^[9]^ Furthermore, animal studies have reported that vit D inhibits cartilage degradation and improves subchondral bone.^[10]^

Deficiencies in 25-hydroxyvitamin D (25(OH)D) are associated with severe knee pain, rapid OA progression,^[11]^ and impaired muscle strength.^[12]^ Therefore, vit D deficiency may contribute to OA pathogenesis,^[13]^ particularly in older adults who are prone to low vitamin levels and have poor physical function.^[14]^ Therefore, vit D supplementation may offer an alternative treatment for improving physical function in this population.

Existing clinical trials on vit D supplementation for knee OA have demonstrated varying results,^[15–20]^ with limited effects on pain and muscle strength and inconsistent findings regarding symptom relief and OA progression. Active vitamin D3 (vit D3) has greater efficacy than inactive forms in improving bone mineral density (BMD) and reducing fracture risk; however, research on its effects on knee OA is limited.^[21]^

Vitamin E (vit E), known for its antioxidant properties, has also been investigated for its potential protective effects against chronic diseases, including OA.^[22,23]^ Different forms of vit E, particularly α- and γ-tocopherol, reduce the levels of pro-inflammatory cytokines and inhibit processes such as prostaglandin E2 synthesis,^[24]^ suggesting a role in modulating inflammation in OA. In knee OA, higher levels of reactive oxygen species in chondrocytes can drive the breakdown of the extracellular matrix, apoptosis, and boost the release of inflammatory cytokines, playing a major role in disease progression.^[25]^ At the cellular level, these studies have also shown that vit E (particularly α-tocopherol) is a fat-soluble antioxidant that suppresses oxidative stress-induced chondrocyte death and matrix metalloproteinase 3 expression.^[26]^ The ability of vit E to reduce oxidative stress and protect cartilage has been confirmed in animal models.^[27]^ However, clinical findings have been inconsistent, with some studies reporting positive effects on joint health and others showing no correlation, or even negative effects.^[28–33]^

Considering the potential effects of vit D3 and vit E, we hypothesized that combining active vit D3 or vit E supplementation with evidence-based lower limb exercise would have additive effects on pain reduction, muscle strength development, performance, and quality of life in patients with knee OA. Therefore, this study aimed to investigate whether the combination of active vit D3 or vit E supplementation with therapeutic lower limb home exercises provides additional benefits over home exercise alone in knee OA management.

## 2. Methods

### 2.1. Study design and participants

This prospective, randomized, placebo-controlled clinical trial was conducted at our medical center in Tokyo, Japan, from June 2018 to October 2021. Women with symptomatic knee OA (Kellgren–Lawrence [KL] grades 2–4) were eligible for inclusion. OA diagnosis was confirmed based on patient history, physical examination, and radiographic assessments. The exclusion criteria were as follows: systemic diseases affecting motor function, psychogenic disorders, or neuromuscular diseases; history of knee surgery; ongoing osteoporosis treatment at other hospitals; history of primary hypercalcemia, renal failure, or neurological conditions (e.g., Parkinson disease or prior stroke); and inability to perform physical activities.

After obtaining approval from our Institutional Review Board (certified review board no. 3180012), the study was conducted in accordance with the principles of the Declaration of Helsinki. The paper adheres to Consolidated Standards of Reporting Trials guidelines.

This clinical trial is a preliminary clinical study (pilot study) to confirm the study’s prudence and clinical outcomes in advance and to provide essential data for planned future substantive research. The sample size for the pilot study is not based on statistical power but is guided by the consideration to fulfill the primary aims of the study and practical feasibility.^[34]^

Thus, there were no specific calculation criteria for the number of participants, and 88 participants were randomly assigned to the control and experimental groups. Of the initially enrolled participants, 8 were excluded from the final analysis owing to loss to follow-up. As a result, 80 patients with knee OA completed the study and were included in the final analysis (Fig. 1).

**Figure 1.**
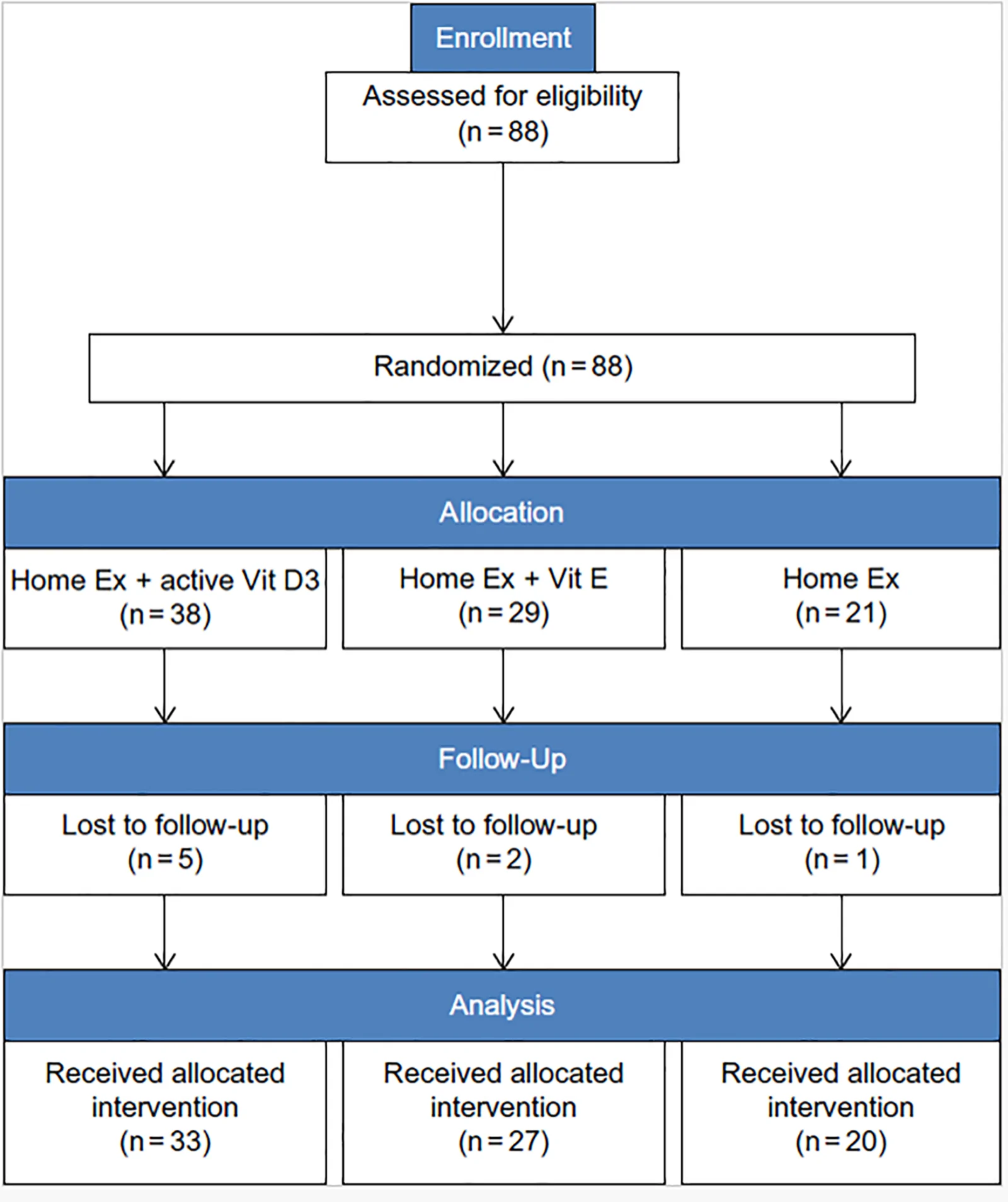
CONSORT flow diagram. CONSORT = Consolidated Standards of Reporting Trials, Ex = exercise, Vit D3 = vitamin D3, Vit E = vitamin E.

The study was registered in the Japan Registry of Clinical Trials (jRCTs 031180357), and all participants provided written informed consent before participation.

An independent researcher randomly assigned participants to 1 of 3 groups using a computer-generated list: therapeutic lower limb home exercise only (the “Home Ex” group), home exercise combined with active vit D3 administration (the “Home Ex **+** active vit D3” group), and home exercise combined with vit E administration (the “Home Ex **+** vit E” group). Data were collected at baseline and after 24 weeks to assess and compare therapeutic effects between the 3 groups.

### 2.2. Interventions

#### 2.2.1. Isometric muscle exercises of the lower limbs

All participants were instructed to perform isometric muscle exercises of the lower limbs as part of their home exercise regimen. Our previous prospective study that compared therapeutic home exercises with intra-articular hyaluronic acid injections in women with OA showed comparable improvements in outcomes and reported that home exercises are an effective initial treatment for knee OA owing to their safety and noninvasiveness.^[35]^ Therefore, in this study, we selected relatively simple home exercises that involve isometric contractions. Physical therapists provided instructions and a brochure detailing the exercises, and the participants received additional guidance from doctors during outpatient clinic visits before the clinical trials. The home exercise program consisted of 3 exercises: straight leg raising, hip abduction, and hip adduction, which were performed twice daily (once in the morning and once in the evening). Each exercise involved holding muscle contractions for 5 to 10 seconds, followed by 5 seconds of relaxation, and repeating these steps 20 times per exercise cycle. In the straight leg raising exercise, participants lay on their backs, raised the involved leg straight up, and kept the ankle 10 cm above the floor for 5 seconds before slowly lowering it. The hip abduction exercise involved participants lying on their side with the affected leg on top, slowly lifting the affected leg to a horizontal position, holding it for 5 seconds, and slowly lowering it. In the hip adduction exercise, the participants sat on the edge of a table or high chair with their knees bent, squeezed a ball between their knees, held it for 5 seconds, and then slowly opened their knees. The frequency and intensity of these exercises were monitored using participant diaries, and regular checkups were conducted to verify adherence. During outpatient visits, doctors ensured that the participants performed their home exercises daily.

#### 2.2.2. Active vit D3 administration

Eldecalcitol, an active vit D3 analog (EDIROL: eldecalcitol 0.75 μg <30 IU>/CHUGAI Pharmaceuticals Co.), was used in this study. Eldecalcitol increases bone density and reduces vertebral fractures more effectively than other active vit D3 analogs, such as alfacalcidol,^[36–38]^ and is commonly prescribed for osteoporosis treatment in Japan. Eldecalcitol has favorable safety and efficacy profiles for preventing and treating osteoporosis. For this study, participants in the active vit D3 group were instructed to take 1 capsule (30 IU) daily for 6 months after breakfast. This dose is consistent with the standard regimen for osteoporosis prevention and treatment in Japan. This study aimed to assess the additive effects of active vit D3 combined with therapeutic home exercises for knee OA.

#### 2.2.3. Vit E administration

Vit E (Juvela: tocopherol acetate 50 mg <50 IU>, Eisai Pharmaceuticals Co.) is commonly prescribed in Japan to treat peripheral circulation disorders such as arteriosclerosis and intermittent claudication. In this study, participants in the vit E group were instructed to take 2 capsules (100-IU total) of vit E daily, either after breakfast or dinner, for 6 months. This study aimed to evaluate the additive effects of vit E combined with home-based therapeutic exercises for knee OA.

### 2.3. Evaluation

The primary outcome measure was the Japanese knee osteoarthritis measure (JKOM). Secondary outcomes included performance on the short-form health survey (SF-36), grip strength (GS), quadriceps muscle strength (QMS), physical performance (10-m walk test [10 MWT]), biochemical markers (blood urea nitrogen, creatinine, and calcium phosphorus), inflammatory markers (high-sensitivity C-reactive protein [hs-CRP] and transforming growth factor-beta [TGF-β]), BMD, and body composition.

Additionally, the study monitored potential adverse effects, including hypercalcemia and renal failure, and recorded prescribed analgesics (cyclooxygenase-2 inhibitors) as rescue medications. Final evaluation was performed over a 24-week follow-up period.

#### 2.3.1. Knee function and health-related quality of life (HRQOL)

Knee function and quality of life were assessed using JKOM^[39]^ and SF-36, respectively.^[40]^ JKOM includes a 100-mm visual analog scale (VAS) and 4 subscales: pain and stiffness (8 questions, 32-point scale), activities of daily living (10 questions, 40-point scale), general activities (5 questions, 20-point scale), and health conditions (2 questions, 8-point scale). In this study, only the JKOM VAS score, pain and stiffness, activities of daily living, and total score were used, with lower scores indicating better knee function (response: 0–4 points; 0 indicates no pain or difficulty, and 4 represents extreme pain or difficulty).

The SF-36 evaluates HRQOL, including the physical component summary, mental component summary, and role/social component summary, with scores ranging from 0 to 100; higher scores indicate better quality of life and well-being.^[40]^ These assessments were performed at baseline and at the 6-month follow-up.

#### 2.3.2. Radiography

At baseline, radiographic assessments included fully extended weight-bearing anteroposterior tibiofemoral and skyline patellar views. Disease severity was measured using the KL grade, with joint space narrowing serving as the key indicator. The smallest medial joint space width of the index knee was measured in the anteroposterior view. The femorotibial angle was calculated using the method described by Moreland et al.^[41]^

#### 2.3.3. Muscle strength and physical performance

Muscle strength and physical performance were assessed by physical therapists at baseline and 6 months after treatment. GS (kg) was measured using an analog grip dynamometer (T.K.K.5001Grip-A, Takei Scientific Instruments Co., Ltd.), with 2 alternate measurements on the dominant hand, and the mean value was recorded.^[42]^ QMS (kgf) was measured using a handheld μ-Tas F-1 dynamometer (ANIMA Corporation).^[43]^ Each participant sat with their knees flexed at 90° and pressed against a dynamometer placed 5 cm above the transmalleolar axis. They held the maximum force position for 5 seconds.^[41]^ The 10 MWT (m/s) was used to evaluate physical performance by measuring the time required to walk 10 m.^[44]^

#### 2.3.4. Biochemical tests including inflammatory markers

Biochemical tests were performed at baseline and after 6 months. Fasting venous blood samples were collected in the early morning and centrifuged, and the serum and plasma were stored at −70°C until analysis. Serum blood urea nitrogen, creatine, and calcium levels were measured using a LABOSPECT 008 autoanalyzer (Hitachi High-Technologies Ltd.). Serum 25(OH)D, hs-CRP, and TGF-β levels were measured by SRL, Inc.. Vit D status was classified as deficient (<20 ng/mL), insufficient (20–29 ng/mL), or sufficient (≥30 ng/mL), according to Japan Endocrine Society guidelines.

#### 2.3.5. Body composition

Body mass index (BMI) was calculated by dividing the individual’s weight (kg) by the square of their height (m). BMD measurements of the lumbar spine and femoral neck were performed using a Prodigy dual X-ray absorptiometry system (GE Healthcare Japan Co., Ltd.). Additionally, skeletal muscle mass index (kg/m^2^) and body fat percentage were evaluated using dual-energy X-ray absorptiometry. Skeletal muscle mass index was calculated by summing the skeletal muscle masses of the upper and lower limbs (kg).

### 2.4. Statistical analysis

SAS software (version 9.4; Cary) was used for data analysis. The results are presented as mean (standard deviation). Comparisons between baseline and 24-week follow-up data within each group were assessed using a paired *t* test for parametric data and the Wilcoxon signed-rank test for nonparametric data. Analysis of variance was used to compare the percentage change between baseline and 6-months between the 3 groups, and multiple comparison tests were used to compare the effects of the combination of active vit D3 or vit E supplementation with therapeutic lower limb home exercise to home exercise alone. Statistical significance was set at *P* < .05.

A linear regression analysis was conducted with the JKOM total score as the dependent variable and the study group (Home Ex + active vit D3, Home Ex + vit E, or Home Ex) as the primary independent variable. Covariates, including age, BMI, and KL grades, were selected based on prior studies and clinical judgment and were entered into a multivariate regression model. Analysis of covariance was used to compare the groups, adjusting for age, BMI, and KL grade with respect to the total JKOM score at 6 months.

## 3. Results

The Consolidated Standards of Reporting Trials 2010 flow diagram for this study is shown in Figure 1. Eighty-eight postmenopausal women (mean age 71.9 ± 5.6 years; range 55–80) were enrolled in the study. Five participants in the Home Ex + active vit D3 group, 2 in the Home Ex + vit E group, and 1 in the Home Ex group were lost to follow-up. As a result, 80 participants (91%) completed the follow-up: 33 in the Home Ex + active vit D3 group, 27 in the Home Ex + vit E group, and 20 in the Home Ex group. Participants were analyzed using a per-protocol approach. Regarding disease severity, 43% (34/80) had KL grade 2, 31% (25/80) had KL grade 3, and 26% (21/80) had KL grade 4. None of the tested parameters showed significant differences between the 3 groups at baseline (Table 1).

**Table 1:** Demographic characteristics of the groups.

	Home Ex + active vit D3	Home Ex + vit E	Home Ex	*P* value
n	33	27	20	
Age (yr)	72.41 (5.32)	70.48 (5.08)	70.05 (6.17)	.227
Height (cm)	152.1 (6.38)	154.2 (4.46)	155.0 (5.86)	.210
Body weight (kg)	55.95 (9.542)	59.83 (10.7)	57.76 (8.79)	.533
BMI (kg/m^2^)	24.20 (3.68)	24.96 (3.71)	23.56 (3.52)	.537
KL grade 2	14	10	10	–
KL grade 3	9	7	9	–
KL grade 4	10	10	1	–
JSW (mm)	2.01 (1.51)	1.73 (1.45)	2.06 (1.16)	.680
FTA (degree)	180.4 (5.98)	81.0 (4.05)	180.3 (4.71)	.856

Values are shown as mean (SD), *P* values obtained using ANOVA tests.

ANOVA = analysis of variance, BMI = body mass index, cm = centimeter, Ex = exercise, FTA = femorotibial angle, JSW = joint space width, KL = Kellgren–Lawrence, SD = standard deviation, vit D3 = vitamin D3, vit E = vitamin E.

### 3.1. Effects on symptoms and HRQOL

After 6 months, the VAS and JKOM total scores showed significant improvement from the baseline values in all 3 groups (*P* ≤ .016). Significant improvements were observed in the physical component summary of SF-36 (*P* = .002) in the Home Ex + active vit D3 group after 6 months. However, neither the 6-month scores nor the baseline-to-6-month percentage change for the JKOM and SF-36 were significantly different between the 3 groups (Table 2).

**Table 2:** Effects of active vit D3 or vit E intervention on JKOM, SF-36, and physical performance.

Outcome		Home Ex + active vit D3	Home Ex + vit E	Home Ex	Percentage change from baseline to 6 months between the 3 groups
JKOM					*P* value^‡^
VAS	Baseline	53.7 (22.6)	59.5 (27.1)	53.5 (25.5)	
	6 mo	30.3 (21.8)	34.1 (22.67)	29.8 (22.8)	
	*P* value^†^	<.001*	<.001*	.016*	.809
Pain, stiffness	Baseline	13.8 (6.44)	14.5 (7.12)	13.5 (6.78)	
	6 mo	9.83 (6.03)	9.92 (6.76)	7.25 (3.91)	
	*P* value^†^	<.001*	<.001*	.001*	.492
ADL	Baseline	11.3 (6.72)	10.3 (7.02)	10.2 (6.74)	
	6 mo	6.48 (5.71)	7.17 (7.00)	4.65 (3.62)	
	*P* value^†^	<.001*	.002*	<.001*	.818
Total score	Baseline	35.2 (17.5)	34.9 (18.3)	33.6 (17.1)	
	6 mo	21.7 (14.8)	23.8 (16.4)	17.0 (8.65)	
	*P* value^†^	<.001*	<.001*	.001*	.634
SF-36					
PCS	Baseline	34.0 (10.03)	35.6 (7.28)	35.7 (7.04)	
	6 mo	39.7 (9.58)	38.3 (9.83)	39.7 (8.32)	
	*P* value^†^	.002*	.127	.157	.397
MCS	Baseline	55.0 (8.08)	55.3 (8.27)	55.7 (8.97)	
	6 mo	55.7 (9.01)	56.2 (9.73)	56.0 (6.92)	
	*P* value^†^	.597	.653	.915	.985
RCS	Baseline	43.7 (13.17)	47.9 (9.84)	42.9 (16.19)	
	6 mo	43.5 (11.01)	38.3 (9.83)	47.1 (14.89)	
	*P* value^†^	.112	.096	.329	.648
Physical performance					
QMS, kgf	Baseline	15.1 (5.33)	15.6 (5.29)	15.9 (3.57)	
	6 mo	18.0 (5.65)	17.9 (6.63)	20.7 (5.76)	
	*P* value^†^	.001*	.003*	<.001*	.297
GS, kg	Baseline	17.5 (4.19)	17.7 (4.53)	18.1 (5.31)	
	6 mo	19.3 (4.68)	19.0 (3.58)	18.1 (4.21)	
	*P* value^†^	.002*	.081	.988	.617
10 MWT, s	Baseline	10.2 (3.28)	9.67 (1.89)	9.19 (1.77)	
	6 mo	8.92 (2.18)	8.44 (1.22)	7.76 (1.11)	
	*P* value^†^	.007*	<.001*	<.001*	.423

Values are shown as mean (SD), and *P* values^†^ were obtained using a paired *t* test or Wilcoxon signed-rank test.

*P* values^‡^ were obtained using ANOVA. *P* < .05.

10 MWT = 10-m walk speed test, ANOVA = analysis of variance, EX = exercise, GS = grip strength, JKOM = Japanese knee osteoarthritis measure, MCS = mental component summary, PCS = physical component summary, QMS = quadriceps muscle strength, RCS = role/social component summary, SD = standard deviation, SF-36 = short-form health survey-36, vit D3 = vitamin D3, vit E = vitamin E.

* Significant difference.

### 3.2. Effects on muscle strength and physical performance

Significant improvements were observed in QMS (*P* < .003) and 10 MWT (*P* < .007) in all 3 groups after 6 months. In contrast, significant increases in GS from baseline to 6 months were noted in the Home Ex + active vit D3 group (*P* = .002). However, neither the 6-month values nor the baseline-to-6 month change rate (%) in QMS, GS, and 10 MWT values were significantly different between the 3 groups (Table 2).

### 3.3. Effects on biochemical tests, including inflammatory markers

No significant differences were observed in the circulating concentrations of inflammatory markers (hs-CRP and TGF-β) or baseline-to-6-month change rate (%) between the 3 groups at 6 months; however, decreases in inflammatory markers from baseline to 6 months were noted in the Home Ex + vit E group (hs-CRP: *P* = .027, TGF-β: *P* = .035) (Table 3). During active vit D3 administration, none of the participants developed hypercalcemia (Ca > 10.5 mg/dL).

**Table 3:** Effects of active vit D3 or vit E intervention on biochemical markers and body composition.

Outcome		Home Ex + active vit D3	Home Ex + vit E	Home Ex	Percentage change from baseline to 6 months between the 3 groups
Inflammatory markers					*P* values^‡^
hs-CRP, ng/mL	Baseline	767.7 (1051)	1117.0 (1009)	8056 (2695)	
	6 mo	890.2 (1091)	817.5 (715.9)	1798 (4071)	
	*P* value^†^	.916	.027*	.712	.267
TGF-β,ng/mL	Baseline	30.04 (12.23)	35.47 (9.520)	28.20 (12.06)	
	6 mo	26.31 (11.12)	29.14 (11.13)	25.17 (14.24)	
	*P* value^†^	.377	.035*	.368	.558
Biochemical tests					
25(OH)D, ng/mL	Baseline	16.27 (5.57)	16.21 (5.43)	16.91 (6.26)	
	6 mo	16.76 (4.09)	14.54 (4.14)	16.66 (5.99)	
	*P* value^†^	.486	.068	.670	.122
BUN, mg/dL	Baseline	16.9 (4.82)	16.2 (3.75)	16.7 (3.59)	
	6 mo	18.2 (5.30)	15.7 (3.39)	16.7 (3.69)	
	*P* value^†^	.307	.704	.462	.676
Crea, mg/dL	Baseline	0.92 (1.66)	0.64 (0.11)	0.60 (0.12)	
	6 mo	1.01 (1.90)	0.65 (0.13)	0.61 (0.13)	
	*P* value^†^	.096	.600	.855	.338
Ca, mg/dL	Baseline	9.6 (0.39)	9.79 (0.39)	9.7 (0.33)	
	6 mo	9.68 (0.33)	9.70 (0.40)	9.63 (0.31)	
	*P* value^†^	.039*	.198	.340	.163
Body composition					
LS-BMD, %	Baseline	82.87 (14.0)	93.18 (21.35)	90.64 (16.37)	
	6 mo	84.39 (13.29)	91.82 (19.45)	90.92 (17.67)	
	*P* value^†^	.005*	.303	.804	.082
FN-BMD, %	Baseline	81.09 (13.47)	83.45 (9.87)	87.22 (17.45)	
	6 mo	82.00 (12.99)	81.36 (8.18)	85.09 (17.31)	
	*P* value^†^	.105	.011*	.681	.003*
Whole YAM, %	Baseline	92.88 (8.22)	95.75 (1.71)	96.38 (10.40)	
	6 mo	92.63 (7.98)	94.75 (1.26)	95.38 (10.66)	
	*P* value^†^	.563	.092	.033	.320
SMI, kg/m^2^	Baseline	6.02 (0.68)	5.81 (0.59)	6.27 (0.59)	
	6 mo	6.01 (0.76)	5.85 (0.67)	6.29 (0.70)	
	*P* value^†^	.827	.701	.801	.893
Fat mass, g	Baseline	21,021 (6186)	26,328 (5475)	20,457 (6857)	
	6 mo	20,291 (6636)	25,628 (5622)	19,740 (6667)	
	*P* value^†^	.135	.111	.090	.936
Nonfat mass, g	Baseline	33,254 (4001)	32,562 (4268)	34,929 (3611)	
	6 mo	33,855 (4410)	33,369 (4108)	35,578 (4394)	
	*P* value^†^	.110	.108	.134	0.839
Body fat percentage, %	Baseline	38.17 (5.65)	44.48 (2.58)	43.13 (3.16)	
	6 mo	36.79 (6.49)	43.13 (3.16)	35.03 (8.41)	
	*P* value^†^	.037*	.129	.045*	.944

Values are shown as mean (SD), and *P* values^†^ were obtained using a paired *t* test or Wilcoxon signed-rank test.

*P* values^‡^ were obtained using ANOVA. *P* < .05.

ANOVA = analysis of variance, BUN = blood urea nitrogen, Ca = calcium, Crea = creatine, Ex = exercise, FN-BMD = bone mineral density of the femoral neck, hs-CRP = high-sensitivity C-reactive protein, LS-BMD = bone mineral density of the lumbar spine, SD = standard deviation, SMI = skeletal muscle mass index, TGF-β = transforming growth factor-beta, vit D3 = vitamin D3, vit E = vitamin E, YAM = young adult mean.

* Significant difference.

### 3.4. Effects on body composition

No significant differences were observed in body composition percentage change from baseline to 6 months between the 3 groups, except for femoral neck-BMD (*P* = .003). Furthermore, regarding lumbar spine-BMD, no significant difference was observed in the multiple comparison test between the group that received active vit D3 or vit E supplementation with lower limb home exercise and the group that received home exercise alone. After 6 months, body fat percentage showed significant positive effects in the Home Ex + active vit D3 and Home Ex groups (*P* = .037, *P* = .045), with no significant differences in the rates of change (%) from baseline to 6 months between the 3 groups (Table 3).

### 3.5. Multivariable analysis

No significant additive effects were found in the Home Ex + active vit D3 and Home Ex + vit E groups, even after adjusting for several covariates. Age (*P* = .877), BMI (*P* = .698), KL grade 2 (*P* = .496), and KL grade 3 (*P* = .310) made only minor contributions to the regression model (Table 4).

**Table 4:** Multivariate linear analysis using JKOM total score as the objective variable.

Independent variables	Adjusted linear regression		
Categorical variables	Beta value	95% CI	*P*
Intercept	23.05	−28.28 to 74.38	.373
Home Ex + active vit D_3_	6.67	−1.99 to 15.33	.129
Home Ex + vit E	8.67	−0.28 to 17.62	.057
Home Ex	–	–	–
Covariates age	−0.05	−0.66 to 0.57	.877
BMI	−0.18	−1.13 to 0.76	.698
KL2	−3.00	−11.75 to 13.79	.496
KL3	4.67	−4.44 to 5.75	.310
KL4	–	–	–

Objective variable: JKOM total score. Explanatory variables: Home Ex + active vit D3, Home Ex + vit E, and Home Ex.

Adjusted model included: age (as continuous), BMI (as continuous), and KL grade (degrees).

Beta = regression coefficients, BMI = body mass index, CI = confidence interval, Ex = exercise, JKOM = Japanese knee osteoarthritis measure, KL = Kellgren–Lawrence grades, vit D3 = vitamin D3, vit E = vitamin E.

## 4. Discussion

The addition of active vit D3 or vit E to lower limb home exercises for 6 months did not yield significant additional benefits for symptoms, muscle strength, or physical performance in patients with knee OA. In other words, although the addition of vit D or E supplementation to exercise therapy for patients with knee OA resulted in improvements in pain, physical function, and muscle strength in all 3 groups, no clear additive effect was observed in the supplementation groups compared to the exercise-only group. However, supplementary outcomes demonstrated characteristic trends: vit E was associated with improved inflammatory markers, vit D was associated with favorable changes in physical health and bone density, and both vit D and exercise reduced body fat. Although these differences did not reach statistical significance, they suggest nutrient-specific physiological effects that may be more pronounced in selected patient populations. This result is consistent with the limited evidence on the efficacy of nutritional supplements, such as active vit D3 and vit E, in knee OA when combined with exercise.

Several studies have shown that exercise intervention has beneficial effects on knee OA, bone metabolism, and gut microbiota, particularly in older adults.^[45–48]^ Two prospective studies that compared home exercises with intra-articular hyaluronate injections in female patients with OA showed equal improvements in outcomes, suggesting that home exercise is an effective initial treatment for knee OA owing to its safety, low cost, and noninvasiveness.^[35,49]^ Although home exercises are beneficial, evidence for the efficacy of vitamin supplementation in knee OA is unclear. Eldecalcitol, an active vit D3 analog, has been of particular interest because, unlike the inactive forms of vit D, it directly affects target organs. To the best of our knowledge, this study is the 1st to examine the effects of active vit D3 (eldecalcitol) on knee OA symptoms; however, it did not show significant improvements in symptoms, muscle strength, or physical performance compared with exercise alone.

In contrast, previous studies on vit D supplementation in patients with knee OA have reported inconsistent results. For instance, randomized clinical trials on cholecalciferol (vit D3) supplementation (800–2000 IU/d) conducted over 2 to 3 years did not show significant improvements in Western Ontario and McMaster Universities Osteoarthritis Index (WOMAC) scores.^[15,16]^ However, a meta-analysis by Gao et al^[17]^ suggested that vit D supplementation improved WOMAC pain and function. Although our study did not observe any significant benefits of active vit D3 supplementation, it aligns with these mixed findings.

Previous randomized clinical trials in which vit E was administered at 500 IU/d did not show a significant improvement in WOMAC scores.^[31,33]^ One study reported improvement in postoperative WOMAC scores in patients with knee OA who received 400 IU of vit E for 2 months before undergoing total knee arthroplasty.^[32]^ We found no significant additive effects of vit E supplementation in combination with lower limb home exercises on knee OA symptoms.

There is limited evidence regarding the effects of vit D supplementation on muscle strength and physical performance in patients with knee OA. Some studies on vit D_2_ (5400 IU/d for 6 months or 1000 IU/d for 1 year) in patients with knee OA showed improvements in quality of life, GS, timed up-and-go test, and hip muscle strength, particularly in older adults with vit D insufficiency.^[18,19]^ A randomized study of 686 older Australian women found no changes in GS and timed up-and-go test scores with vit D3 supplementation (150,000 IU every 3 months) over 9 months.^[18]^ In our study, the Home Ex + active vit D3 group showed a modest improvement in GS, but no significant differences were observed when compared to the Home Ex group.

In our study, the Home Ex + vit E group showed significant improvements in QMS and 10 MWT values after 6 months, but the changes from baseline to 6 months were not significantly different between the 3 groups. Although some studies have suggested the potential antioxidant and anti-inflammatory benefits of vit E in older adults,^[32]^ our study did not reveal significant differences between the groups, despite a trend toward reduced inflammatory markers in the Home Ex + vit E group. For example, 1 study investigated the effect of vit E supplementation in older adults with knee OA and found that while it reduced antioxidant enzyme activity (superoxide dismutase and erythrocyte malondialdehyde), it did not significantly affect systemic inflammatory markers such as CRP and IL-6.^[50]^ Similarly, we observed a larger decrease in inflammatory markers from baseline to 6 months in the Home Ex + vit E group than in the other groups, although this difference was not significant.

We focused on vit D and vit E as research targets because both nutrients are mechanistically linked to key pathophysiological processes in OA, including cartilage metabolism, muscle function, oxidative stress, and inflammation, and exert well-documented systemic effects across multiple organ systems. Vit D acts as a hormone influencing musculoskeletal, immune, metabolic, and cardiovascular functions, with established roles in bone health, muscle strength, and immune regulation, and is particularly relevant for OA. Vit E, a major lipid-soluble antioxidant, reduces oxidative stress and modulates inflammatory pathways across the cardiovascular, neurological, dermatological, and immune systems. Considering the importance of oxidative stress and inflammation in OA, the inclusion of vit D and vit E in therapies for this population is justified based on their OA-related mechanisms and broader physiological significance.

The lack of significant findings regarding the additive effects of vitamins alongside therapeutic lower limb home exercises may be due to several factors, including dose, duration, and patient characteristics. The doses of active vit D3 (30 IU/d) and vit E (100 IU/d) used in this study are lower than those commonly administered in Western countries. Active vit D3, an approved prescription drug in Japan, is typically administered at a dose of 30 IU/d to treat osteoporosis.^[36–38]^ In contrast, larger doses of vit D3 have been used in Western clinical trials. Similarly, the prescribed vit E dose of 100 IU/d in Japan is approximately 20% to 25% of the amount used in Western supplementation studies. These dosage differences may explain why the effects observed in this study are less pronounced than those reported in international studies.

Another factor was the relatively short duration of the study. Our primary objective was to evaluate the additive effect of vitamins in combination with therapeutic lower limb home exercises rather than the impact of vitamins alone. Since therapeutic home exercise is voluntary, a longer study period might have led to participant fatigue or decreased motivation, which is why we limited the study duration to 6 months. Additionally, the baseline serum 25(OH)D levels of the participants were relatively low (mean 17.87 ng/mL), suggesting a deficiency in vit D, as the average serum 25(OH)D level in healthy Japanese individuals was 20.3 ng/mL.^[51]^ Given the slow nature of the potential benefits of vitamin supplementation, a longer follow-up period is necessary to observe more substantial effects.

Furthermore, the study sample consisted solely of Japanese women, who tend to have lower BMI and serum 25(OH)D concentrations than those in European and North American populations. This raises questions regarding the generalizability of our findings to other ethnic groups. Additionally, the effects of aging, including estrogen depletion, could influence the body’s ability to activate vitamins efficiently, which may have contributed to the lack of significant effects. Further research is needed to explore the metabolic pathways involved in vits D3 and E supplementation and their effects across different ages, sexes, and ethnicities.

The strengths of this study include its prospective, randomized, controlled, interventional design with concealed assignments and high participant adherence (91% follow-up rate). This is the 1st study to explore the additive effects of active vit D3 or E combined with home exercise therapy on knee OA in a Japanese population. Another unique aspect of this study is that both vitamins were tested together, whereas most previous studies focused on only 1 vitamin.

However, this study had some limitations. It included only women, and we did not assess markers of oxidative damage or serum vit E levels, limiting our ability to evaluate the antioxidant effects of vit E. Additionally, the 6-month study duration may not have been long enough to capture long-term drug efficacy. Furthermore, the total number of participants included in the final analysis of this study appeared too small to adequately assess drug efficacy. These results do not clarify whether vitamin supplementation is effective, indicating the need for long-term studies with larger sample sizes.

Future research should identify responsive subgroups (including those with vit D deficiency, elevated oxidative stress, reduced bone density, and high levels of inflammation), optimize dosage and duration, examine interactions with different exercise modalities, investigate mechanisms using biochemical markers, and evaluate long-term structural outcomes. These directions will clarify the clinical relevance of the observed supplementary effects and support the development of more individualized treatment strategies.

## 5. Conclusion

Adding active vit D3 or vit E to therapeutic lower limb home exercises for 6 months did not yield significant additive benefits for symptoms, muscle strength, or physical performance in patients with knee OA.

We confirmed that lower limb exercise therapy plays a key role in the nondrug treatment of OA. This study suggests that the effectiveness of lower limb exercise therapy combined with nutritional supplementation likely depends on the patient’s background and clinical pathology, and that more precise individualized studies are needed.

## Acknowledgments

We thank Daisuke Koide, PhD, MD, for statistical advice. Editorial support, in the form of medical writing, copyediting, and fact-checking, was provided by Editage, Cactus Communications.

## Author contributions

**Conceptualization:** Hiroaki Kanazawa, Hisashi Kurosawa.

**Data curation:** Hiroaki Kanazawa, Hisashi Kurosawa.

**Formal analysis:** Hiroaki Kanazawa, Masato Koike.

**Funding acquisition:** Hisashi Kurosawa.

**Investigation:** Hiroaki Kanazawa, Hisashi Kurosawa.

**Methodology:** Hisashi Kurosawa.

**Project administration:** Kei Miyagawa, Yu Ozaki.

**Software:** Masashi Nagao, Masato Koike.

**Supervision:** Hisashi Kurosawa, Yoshiyuki Iwase, Muneaki Ishijima.

**Writing – original draft:** Hiroaki Kanazawa.

**Writing – review & editing:** Masashi Nagao, Masato Koike.
